# Supplementary material for: Impact of Maternal Metabolic Status on Human Milk Oligosaccharide Composition: A Population-Based Cross-Sectional Study in Central South China
Source: Nutrients. 2025 Apr 28;17(9):1480. doi: 10.3390/nu17091480 (PMC12073883; doi:10.3390/nu17091480)
Supplement: Supplementary file 1 [file nutrients-17-01480-s001.zip › nutrients-3585946-supplementary.pdf]

Table S1. The association of metabolic factors with neutral fucosylated HMOs among non-secretor mothers by linear regression analysis

	2′-FL				3-FL				LNFP I				LNDFH II			
	β(95%CI)			P	β(95%CI)			P	β(95%CI)			P	β(95%CI)			P
Pre-pregnacy BMI	0.04	(-0.33,	0.41)	0.84	0.17	(-0.20 ,	0.54)	0.37	0.18	(-0.18,	0.55)	0.31	0.04	(-0.33,	0.42)	0.81
Current BMI	0.11	(-0.28,	0.51)	0.56	0.14	(-0.26,	0.54)	0.48	0.29	(-0.09,	0.67)	0.13	0.12	(-0.28,	0.52)	0.54
**TC**	**0.41**	**(0.05,**	**0.78)**	**0.03**	0.22	(-0.17,	0.61)	0.26	0.20	(-0.19,	0.59)	0.30	0.23	(-0.16,	0.62)	0.24
TG	0.27	(-0.07,	0.62)	0.11	-0.25	(-0.60 ,	0.10)	0.16	0.14	(-0.22,	0.49)	0.44	0.02	(-0.35,	0.38)	0.93
HDL	0.04	(-0.32,	0.40)	0.82	0.16	(-0.19,	0.52)	0.36	0.03	(-0.33,	0.38)	0.88	0.05	(-0.31,	0.41)	0.80
**LDL**	**0.55**	**(0.21,**	**0.89)**	**0.00**	0.16	(-0.24,	0.55)	0.42	0.27	(-0.11,	0.66)	0.16	0.25	(-0.14,	0.64)	0.21
GLU	-0.06	(-0.43,	0.32)	0.76	0.07	(-0.31,	0.45)	0.71	0.07	(-0.31,	0.44)	0.72	0.17	(-0.21,	0.54)	0.37
SBP	0.20	(-0.16,	0.56)	0.26	0.21	(-0.15,	0.58)	0.24	**0.37**	**(0.03,**	**0.71)**	**0.03**	0.20	(-0.16,	0.57)	0.26
**DBP**	**0.37**	**(0.05,**	**0.70)**	**0.03**	0.13	(-0.23,	0.49)	0.46	**0.41**	**(0.08,**	**0.73)**	**0.02**	0.26	(-0.09,	0.61)	0.14
HR	0.13	(-0.22,	0.48)	0.46	0.00	(-0.37,	0.36)	0.98	0.10	(-0.26,	0.45)	0.59	0.14	(-0.21,	0.50)	0.42

The model adjusting for maternal age and lactation period;

TC, TG, HDL, LDL and GLU expressed by mmol/L; SBP and DBP expressed by mmHg; HR expressed by bmp;

2′-FL, 3-FL, LNFP I, and LNDFH II expressed by μg/mL

Table S2. The association of metabolic factors with neutral and acidic HMOs among non-secretor mothers by linear regression analysis

Metabolic factors	LNT				LNnT				3′-SL				LSTa			
	β(95%CI)			P	β(95%CI)			P	β(95%CI)			P	β(95%CI)			P
Pre-pregnacy BMI	0.01	(-0.37,	0.39)	0.96	-0.05	(-0.42,	0.33)	0.80	-0.12	(-0.47,	0.23)	0.49	-0.08	(-0.45,	0.29)	0.65
Current BMI	0.09	(-0.31,	0.49)	0.66	0.02	(-0.38,	0.42)	0.90	-0.13	(-0.50,	0.25)	0.49	-0.01	(-0.41,	0.38)	0.94
TC	0.01	(-0.40,	0.41)	0.97	-0.03	(-0.43,	0.37)	0.87	0.04	(-0.33,	0.41)	0.83	-0.08	(-0.47,	0.32)	0.70
TG	0.26	(-0.09,	0.61)	0.14	0.25	(-0.10,	0.60)	0.16	-0.29	(-0.61,	0.03)	0.07	0.26	(-0.08,	0.60)	0.13
HDL	0.03	(-0.33,	0.40)	0.85	0.02	(-0.34,	0.38)	0.92	0.12	(-0.21,	0.45)	0.46	-0.01	(-0.36,	0.35)	0.97
LDL	0.03	(-0.38,	0.43)	0.90	-0.01	(-0.41,	0.39)	0.95	-0.03	(-0.40,	0.35)	0.88	-0.07	(-0.46,	0.32)	0.72
GLU	0.09	(-0.29,	0.47)	0.63	0.15	(-0.23,	0.52)	0.43	0.13	(-0.23,	0.48)	0.47	0.15	(-0.22,	0.52)	0.41
SBP	0.27	(-0.09,	0.64)	0.13	0.23	(-0.13,	0.59)	0.21	0.27	(-0.07,	0.60)	0.11	0.18	(-0.18,	0.54)	0.32
**DBP**	**0.38**	**(0.05,**	**0.72)**	**0.03**	**0.38**	**(0.04,**	**0.71)**	**0.03**	0.24	(-0.09,	0.56)	0.15	0.28	(-0.06,	0.62)	0.10
HR	-0.01	(-0.38,	0.35)	0.94	0.02	(-0.34,	0.38)	0.90	0.01	(-0.33,	0.34)	0.97	-0.06	(-0.41,	0.30)	0.75

The model adjusts for maternal age and lactation period;

TC, TG, HDL, LDL, and GLU expressed by mmol/L; SBP and DBP expressed by mmHg; HR expressed by bmp;

LNT, LNnT, 3′-SL and LSTa expressed by μg/mL

Table S3. The association of metabolic factors with neutral fucosylated HMOs among secretor mothers by linear regression analysis

Metabolic factors	2′-FL				3-FL				LNFP I				LNDFH II			
	β(95%CI)			P	β(95%CI)			P	β(95%CI)			P	β(95%CI)			P
Pre-pregnacy BMI	-0.12	(-0.27,	0.03)	0.12	-0.05	(-0.20,	0.10)	0.51	-0.12	(-0.27,	0.03)	0.13	-0.05	(-0.20,	0.10)	0.52
Current BMI	-0.12	(-0.27,	0.03)	0.11	-0.05	(-0.19,	0.10)	0.55	-0.09	(-0.25,	0.06)	0.22	0.00	(-0.15,	0.16)	0.97
TC	-0.12	(-0.28,	0.04)	0.13	-0.06	(-0.22,	0.09)	0.43	0.04	(-0.12,	0.20)	0.61	0.11	(-0.05,	0.27)	0.18
TG	-0.04	(-0.20,	0.11)	0.60	-0.05	(-0.20,	0.10)	0.51	-0.05	(-0.21,	0.10)	0.48	-0.03	(-0.19,	0.12)	0.67
HDL	-0.02	(-0.17,	0.13)	0.77	0.08	(-0.06,	0.23)	0.26	0.00	(-0.15,	0.15)	0.99	0.00	(-0.15,	0.15)	0.96
LDL	-0.13	(-0.29,	0.03)	0.12	-0.07	(-0.23,	0.08)	0.36	0.03	(-0.13,	0.19)	0.70	0.14	(-0.02,	0.29)	0.09
GLU	-0.04	(-0.20,	0.11)	0.57	0.11	(-0.04,	0.25)	0.15	0.01	(-0.14,	0.16)	0.91	0.08	(-0.07,	0.24)	0.27
SBP	-0.06	(-0.21,	0.09)	0.42	0.01	(-0.13,	0.16)	0.85	-0.02	(-0.17,	0.13)	0.81	0.10	(-0.05,	0.25)	0.21
DBP	0.01	(-0.14,	0.16)	0.88	-0.04	(-0.19,	0.11)	0.62	0.03	(-0.12,	0.18)	0.70	0.09	(-0.07,	0.24)	0.26
HR	0.02	(-0.14,	0.17)	0.84	**0.19**	**(0.04,**	**0.34)**	**0.01**	-0.02	(-0.17,	0.14)	0.84	**0.17**	**(0.02,**	**0.32)**	**0.03**

The model adjusting for maternal age and lactation period;

TC, TG, HDL, LDL and GLU expressed by mmol/L; SBP and DBP expressed by mmHg; HR expressed by bmp;

2′-FL, 3-FL, LNFP I, and LNDFH II expressed by μg/mL

Table S4. The association of metabolic factors with neutral and acidic HMOs among secretor mothers by linear regression analysis

Metabolic factors	LNT				LNnT				3′-SL				LSTa			
	β(95%CI)			P	β(95%CI)			P	β(95%CI)			P	β(95%CI)			P
Pre-pregnacy BMI	0.05	(-0.11,	0.21)	0.53	0.03	(-0.13,	0.19)	0.70	**-0.17**	**(-0.31,**	**-0.03)**	**0.02**	-0.02	(-0.17,	0.13)	0.80
Current BMI	0.04	(-0.11,	0.20)	0.60	0.01	(-0.15,	0.17)	0.89	-0.14	(-0.28,	0.00)	0.06	-0.04	(-0.19,	0.11)	0.60
TC	0.07	(-0.10,	0.23)	0.42	-0.06	(-0.22,	0.11)	0.49	-0.15	(-0.30,	0.01)	0.06	0.01	(-0.15,	0.17)	0.89
TG	-0.08	(-0.24,	0.08)	0.34	-0.05	(-0.20,	0.11)	0.58	-0.05	(-0.19,	0.10)	0.53	-0.13	(-0.28,	0.02)	0.10
HDL	0.03	(-0.13,	0.18)	0.75	-0.04	(-0.19,	0.12)	0.64	0.01	(-0.14,	0.15)	0.92	0.06	(-0.09,	0.21)	0.46
LDL	0.08	(-0.09,	0.24)	0.36	-0.04	(-0.21,	0.12)	0.62	-0.13	(-0.28,	0.02)	0.08	-0.01	(-0.17,	0.15)	0.90
GLU	0.04	(-0.12,	0.19)	0.62	0.06	(-0.10,	0.21)	0.45	0.07	(-0.07,	0.21)	0.34	0.01	(-0.14,	0.16)	0.86
SBP	0.09	(-0.07,	0.24)	0.27	0.13	(-0.03,	0.28)	0.10	-0.05	(-0.19,	0.10)	0.52	0.02	(-0.13,	0.17)	0.78
DBP	0.08	(-0.07,	0.24)	0.30	0.12	(-0.03,	0.28)	0.12	0.03	(-0.11,	0.18)	0.67	0.06	(-0.09,	0.21)	0.46
HR	0.04	(-0.12,	0.19)	0.65	-0.06	(-0.22,	0.09)	0.42	**0.17**	**(0.03,**	**0.32)**	**0.02**	0.01	(-0.14,	0.17)	0.86

The model adjusts for maternal age and lactation period;

TC, TG, HDL, LDL, and GLU expressed by mmol/L; SBP and DBP expressed by mmHg; HR expressed by bmp;

LNT, LNnT, 3′-SL and LSTa expressed by μg/mL

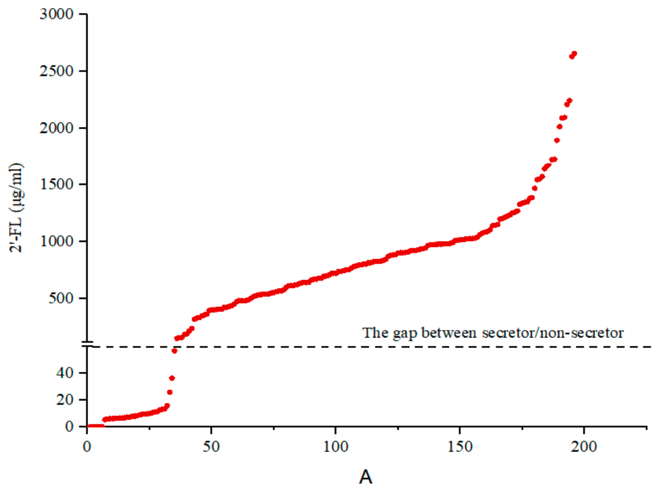

Figure S1. Group of breastmilk HMOs

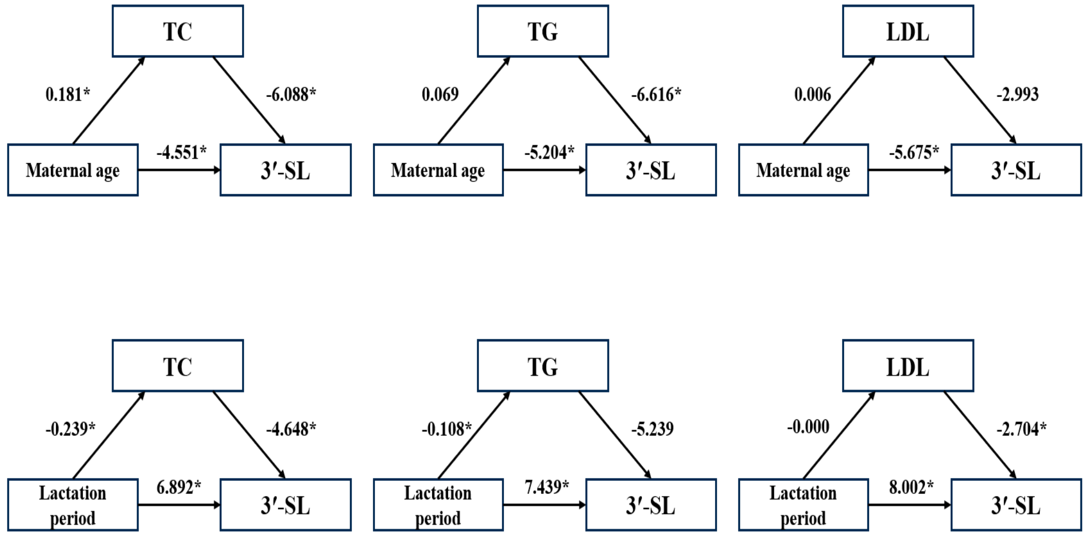

Figure S2. The mediating effect analysis of TC, TG, and LDL on the association of maternal age and lactation period with 3′-SL. HR as covariate was included in mediating effect model. The number above the arrow represented unstandardized regression coefficients; * : P<0.05 for correlation coefficient.
